# Association between tea consumption and frailty among Chinese older adults: A cross-sectional study

**DOI:** 10.3389/fnut.2022.987911

**Published:** 2022-09-20

**Authors:** Shaojie Li, Guanghui Cui, Yongtian Yin, Faqin Lv, Yao Yao

**Affiliations:** ^1^School of Public Health, Peking University, Beijing, China; ^2^China Center for Health Development Studies, Peking University, Beijing, China; ^3^Department of Integrated Traditional Chinese and Western Medicine, Peking University First Hospital, Beijing, China; ^4^Shandong University of Traditional Chinese Medicine, Jinan, China; ^5^Ultrasonic Department, The Third Medical Center of Chinese People's Liberation Army General Hospital, Beijing, China

**Keywords:** tea consumption, frailty, older adults, China, Tilburg Frailty Indicator

## Abstract

**Background:**

Chronic inflammation is considered one of the main mechanisms leading to frailty. It has been demonstrated that tea consumption reduces chronic inflammation. Few epidemiological studies have investigated the association between tea consumption and frailty.

**Objective:**

This study aimed to analyze the association between tea consumption and frailty in Chinese older adults.

**Methods:**

Between March and May 2021, we enrolled 2,144 older adults aged ≥60 years in Jinan City, Shandong Province, China, using multi-stage stratified cluster sampling. We assessed tea consumption and frailty in older adults using the Tilburg Frailty Indicator (TFI) and the frequency of tea consumption, respectively. We applied multiple logistic regression analysis to examine the association between tea consumption and frailty, controlling for a set of potential covariates.

**Results:**

The prevalence of frailty among older Chinese adults was 38.3% (821/2,144). Tea consumption was categorized as daily (30.4%), occasionally (20.9%), and rarely or never (48.7%). As indicated by the fully adjusted model, daily tea consumption was associated with a lower prevalence of frailty (OR = 0.73, 95%CI = 0.57–0.94). However, this association only applied to men, younger older adults aged 60–79 years, rural residents, and regular participants in community activities. In addition, we observed a linear relationship between tea consumption and the prevalence of frailty (*P* for trend = 0.017).

**Conclusions:**

Higher tea consumption was associated with a lower prevalence of frailty in older adults, especially those men, older adults aged 60–79, rural residents, and individuals who regularly participated in community activities. Further longitudinal and experimental studies are needed to determine the causation between tea consumption and frailty.

## Introduction

Frailty is a common geriatric syndrome, that refers to a non-specific condition in which the physiological reserves of an older individual are diminished for a variety of reasons, leading to increased vulnerability and a decreased capacity to withstand stress (1, 2). Currently, there are two main perspectives in assessing frailty (3). One regards frailty as a biological concept mainly evaluated from a single physical function dimension (4). The other believes that frailty has multiple dimensions, including psychological and social fields in addition to the physical field (5). In recent years, this view of multidimensional frailty has been increasingly recognized (6). Meta-analyses have shown that frailty is associated with multiple adverse health outcomes such as premature mortality, disability, and cognitive impairment (7, 8). Moreover, frailty has become a global concern in geriatric health. A meta-analysis of 62 countries and territories showed that the prevalence of frailty and prefrailty was 12% and 46%, respectively, among older adults (9). Data from the China Health and Retirement Longitudinal Study (CHARLS) showed that the prevalence of frailty among older adults has been on the rise, which increased from 18.7% to 28.4% between 2011 and 2015 (10). In addition, CHARLS showed that the incidence of frailty was 60.6/1,000 person-years during an average of 2.1 years of follow-up in 4,939 community-dwelling older adults (11). Given the adverse effects of frailty and its high prevalence and incidence, a growing body of research is exploring factors associated with frailty to provide a scientific basis for developing interventions.

Studying frailty-associated factors from the perspective of pathogenesis is the primary focus of current research. Existing studies have suggested that inflammation may contribute to frailty directly or indirectly through pathophysiological processes such as inhibiting growth factors and by interfering with homeostatic signaling (12–15). Accordingly, studies have explored the relationship between an anti-inflammatory diet (such as the Mediterranean diet) and frailty (16, 17). It has been suggested that these lifestyles may reduce age-related oxidative damage and inflammation, thereby reducing the risk of frailty. In recent years, attention has been drawn to the potential health benefits of tea consumption through its anti-inflammatory qualities. Multiple meta-analyses of observational studies have shown that tea consumption is associated with decreased risks of chronic inflammatory diseases, including cardiovascular disease (18), cancer (19), and depression (20). Existing research has demonstrated that tea contains many polyphenols, especially catechins and their derivatives, which have powerful antioxidant and anti-inflammatory effects *in vivo* (21). Meanwhile, a previous study found that consuming polyphenol plants may slowdown aging and progression of related diseases (22). Moreover, a systematic review found that tea seemed to improve oral microbiota, thus promoting oral health (23) while existing research suggests that oral health may be one of the aspects concerning frailty (24). These findings indicated that tea consumption might be associated with frailty. However, epidemiological studies evaluating the relationship between tea consumption and frailty are limited. To the best of our knowledge, only a few studies have explored the association between green tea consumption and frailty in the older population (25–27). Tea originated in China, and some Chinese people have a habit of drinking tea (28). It is unclear, however, whether tea consumption is associated with a reduced risk of frailty in older adults in China.

Therefore, this study aimed to analyze the association between tea consumption and frailty in older Chinese adults to provide research evidence for potential frailty prevention in the Chinese population. In addition, this study performed a stratified analysis of sex, age, residence, and community activity participation to understand the heterogeneity of the association between tea consumption and frailty.

## Materials and methods

### Participants

We first used the epidemiological sample size estimation formula (29), $\text{n}=\frac{{{\text{Z}}_{\frac{\alpha }{2}}}^{2}\text{P}\left(1\;-\text{ P}\right)}{\delta^{2}}$ to estimate the expected sample size. A previous cross-sectional study conducted in Jinan City, China, showed that the prevalence of frailty among community-dwelling older adults was 34.2% (30). Therefore, in this study using, *P* = 0.342, α = 0.05, μ_α/2_ = 1.96, and δ = 0.03 resulted in a minimum sample size of 961.

**Figure 1:**
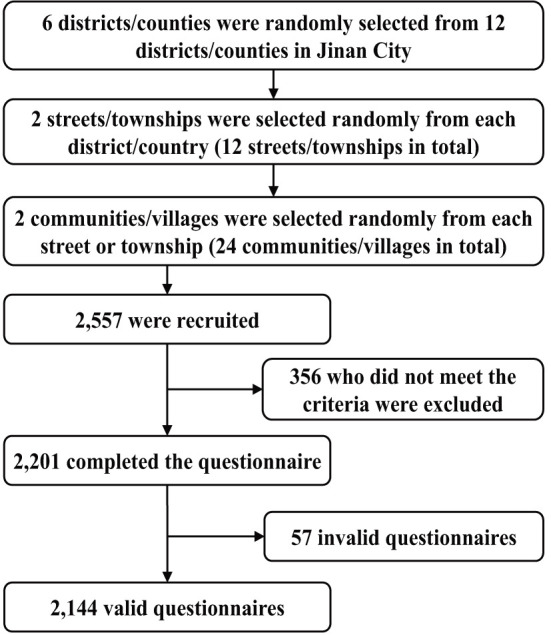
The sampling procedure.

We conducted this cross-sectional survey from March to May 2021 using multi-stage stratified cluster random sampling in Jinan City, Shandong Province. In the first stage, we randomly selected six districts/counties from the 12 districts/counties of Jinan City as the survey area. In the second stage, we randomly selected two townships or streets from the selected six districts/counties. In the third stage, we randomly selected all the older adults in two communities from the selected two townships or streets. The inclusion criteria were as follows: age ≥60 years, living in Jinan for more than 6 months, with no hearing or language impairment, and voluntary participation in the survey. The exclusion criteria were severe diseases, such as dementia (as reported by family members) and complete disability. We recruited 2,557 participants; of these, 356 were excluded because they did not meet the criteria. A total of 2,201 older adults participated in the survey. Among them, 57 participants failed to complete the questionnaire due to reasons such as leaving the survey site. Finally, we received 2,144 completed questionnaires, and the effective survey rate was 97.41%. Figure 1 shows the sampling procedure. All surveys were completed by trained investigators using face-to-face interviews. Written informed consent was obtained from all the participants before the investigation. This study was approved by the Medical Ethics Committee of Xiangya School of Public Health, Central South University (identification code: XYGW-2020-101).

### Measures

#### Tea consumption

We measured tea consumption by asking participants how often they drank tea (approximately 200 ml in each serving) in the past year, and the options included four categories: almost daily, 1–2 times a week, 1–2 times a month, less than once a month or never. Since “1–2 times a month” accounted for a small proportion of our population (< 5%), we combined it and “less than once a month or never” in the data analysis. Finally, according to their answers, the frequency of tea consumption was divided into three categories: rarely or never (“1–2 times a month” and “less than once a month or never”), occasionally (1–2 times a week), and daily (almost daily).

#### Tilburg Frailty Indicator

We used the Chinese version of the Tilburg Frailty Indicator (TFI) (31) to evaluate frailty in older adults. The scale has 15 items, including three dimensions: physical frailty, psychological frailty, and social frailty. Each item was converted into two categories: yes (1) and no (0). A total score of 0 to 15 points was derived from the sum of all items. Frailty is defined as a total score greater than or equal to five. In this study, the Cronbach's alpha for the Chinese version of the TFI was 0.778.

#### Covariates

According to the previous studies (32), we controlled for two sets of covariates, namely sociodemographic characteristics/health status and lifestyle.

Sociodemographic characteristics and health status included age, sex (male vs. female), residence (urban vs. rural), marital status (married vs. unmarried), education level (primary school and below, junior high school, or high school and above), average monthly personal income (< 2,000, 2,000–3,000, or >3,000 yuan), chronic diseases (yes vs. no), body mass index (BMI), and disability. The BMI was calculated using self-reported height and weight. Disability was assessed using the Activities of Daily Living Scale (ADLS). The 14-item scale consists of two subscales: the Physical Self-Maintenance Scale (6 items) and the Instrumental Activities of Daily Living Scale (8 items) (33). Each item was used for a self-assessed four-point Likert-type rating scale (from 1 = no difficulties to 4 = I am not able to do that). The sum of all items gave the total score, ranging from 14 to 56. The higher the score, the more compromised the respondent's ability to perform activities of daily living. A score of 15 or higher was assigned the ADL disability status. In this study, Cronbach's alpha of the ADLS was 0.939.

Lifestyle factors included smoking (yes vs. no), drinking (yes vs. no), vegetable consumption (regular intake vs. occasional or seldom intake), fruit consumption (regular intake vs. occasional or seldom intake), physical activity (regular vs. occasional or seldom participation), and community activity (regular vs. occasional or seldom participation).

### Statistical methods

The Kolmogorov–Smirnov test was used to assess the normal distribution of continuous variables (34). Continuous variables such as age, TFI score, and BMI were described as means ± standard deviation (SD). Categorical variables such as sex and tea consumption were described by frequency and composition ratios. Student's *t*-tests, one-way analyses of variance (ANOVA), the Wilcoxon rank sum test, and the Kruskal–Wallis *H*-test were used to test the differences in age, TFI score, and BMI between categories of tea consumption and frailty, respectively. The chi-squared test was used to test the differences in categorical covariates between tea consumption and frailty categories. We tested the association between tea consumption and frailty using a multiple logistic regression model. Three models were constructed. Model 1 was the raw model. In Model 2, the covariates such as sociodemographic characteristics and health status were included. In Model 3, we included lifestyle factors, including smoking, drinking, vegetable consumption, fruit consumption, physical activity, and community activity. In addition, we performed a stratified analysis to examine the disparity in the association between tea consumption and frailty according to sex, age, and residence. Odds ratios (ORs) and 95% confidence intervals (CIs) were calculated to assess the association between tea consumption and frailty. All statistical analyses were performed using STATA version 16.0 (Stata Corp, College Station, TX, USA).

## Results

### Descriptive statistics

The mean age of the 2,144 older adults enrolled was 72.0 ± 7.0, ranging from 60 to 99 years. A total of 48.7% never or rarely drank tea, 20.9% occasionally drank tea, and 30.4% consumed it daily. The mean TFI score was 4.23 ± 2.91, and the prevalence of frailty was 38.3% (821/2,144). In addition, 1,075 men (50.1%) and 61.3% of the population lived in rural areas, 62.0% had primary education or less, 76.8% were married, and 48.8% had an average monthly income of 2,000 yuan or less. Moreover, compared with participants in the rarely or never drinking tea group, the ones in the daily tea consumption group had significantly lower TFI scores, lower prevalence of frailty, were more likely to live in urban areas, be married, have higher education and monthly income, and had a lower ADL disability rate. In addition, those who consumed tea daily had higher BMIs and were more likely to consume fruits and engage in physical activity. Detailed information regarding the participants' general characteristics is summarized in Table 1.

**Table 1:** Descriptive statistics of participants' characteristics.

**Variables**	**Total sample**	**Tea consumption**				**Frailty**		
		**Rarely or never**	**Occasional**	**Daily**	***P*-value**	**Non-frailty**	**Frailty**	***P*-value**
Total sample, *n* (%)	2,144 (100.0)	1,044 (48.7)	448 (20.9)	652 (30.4)		1,323 (61.7)	821 (38.3)	
TFI								
Total score, Mean ± SD	4.23 ± 2.91	4.60 ± 3.07	4.10 ± 2.83	3.73 ± 2.58	< 0.001	2.33 ± 1.16	7.29 ± 2.16	< 0.001
Physical frailty, Mean ± SD	1.83 ± 2.04	2.05 ± 2.15	1.76 ± 2.00	1.53 ± 1.84	< 0.001	0.62 ± 0.81	3.78 ± 1.91	< 0.001
Psychological frailty, Mean ± SD	1.27 ± 1.16	1.38 ± 1.18	1.25 ± 1.17	1.10 ± 1.11	< 0.001	0.69 ± 0.87	2.20 ± 0.95	< 0.001
Social frailty, Mean ± SD	1.13 ± 0.47	1.17 ± 0.49	1.09 ± 0.44	1.10 ± 0.46	0.002	1.03 ± 0.40	1.31 ± 0.52	< 0.001
≥5, *n* (%)	821 (38.3)	454 (43.5)	162 (36.2)	205 (31.4)	< 0.001	0 (0.0)	821 (100.0)	< 0.001
Age, years, Mean ± SD	72.01 ± 6.96	72.27 ± 7.19	71.84 ± 6.82	71.71 ± 6.68	0.224	70.87 ± 6.37	73.85 ± 7.47	< 0.001
Age group, years, *n* (%)					0.209			< 0.001
60–79	1,822 (85.0)	875 (83.8)	380 (84.8)	567 (87.0)		1,198 (65.8)	624 (34.2)	
Male, *n* (%)	1,075 (50.1)	395 (37.8)	269 (60.0)	411 (63.0)	< 0.001	721 (67.1)	354 (32.9)	< 0.001
Rural residents, *n* (%)	1,315 (61.3)	664 (63.6)	272 (60.7)	379 (58.1)	0.076	740 (56.3)	575 (43.7)	< 0.001
Married	1,646 (76.8)	745 (71.4)	378 (84.4)	523 (80.2)	< 0.001	1,111 (67.5)	535 (32.5)	< 0.001
Educational level, *n* (%)					< 0.001			< 0.001
Primary school and below	1,329 (62.0)	707 (67.7)	252 (56.3)	370 (56.7)		743 (55.9)	586 (44.1)	
Junior high school	494 (23.0)	203 (19.4)	119 (26.6)	172 (26.4)		336 (68.0)	158 (32.0)	
High school and above	321 (15.0)	134 (12.8)	77 (17.2)	110 (16.9)		244 (76.0)	77 (24.0)	
Average monthly personal					< 0.001			< 0.001
income (RMB), *n* (%)							
< 2,000	1,053 (49.1)	579 (55.5)	182 (40.6)	292 (44.8)		524 (49.8)	529 (50.2)	
2,000–3,000	720 (33.6)	338 (32.4)	173 (38.6)	209 (32.1)		512 (71.1)	208 (28.9)	
>3,000	371 (17.3)	127 (12.2)	93 (20.8)	151 (23.2)		287 (77.4)	84 (22.6)	
Chronic disease, *n* (%)	1,509 (70.4)	744 (71.3)	299 (66.7)	466 (71.5)	0.164	830 (55.0)	679 (45.0)	< 0.001
ADL disability, *n* (%)	951 (44.4)	514 (49.2)	180 (40.2)	257 (39.4)	< 0.001	383 (40.3)	568 (59.7)	< 0.001
Body mass index, Mean ± SD	23.19 ± 3.43	22.96 ± 3.51	23.27 ± 3.37	23.49 ± 3.33	0.006	23.28 ± 3.19	23.04 ± 3.78	0.123
Smoking, *n* (%)	636 (29.7)	311 (29.8)	124 (27.7)	201 (30.8)	0.528	320 (50.3)	316 (49.7)	< 0.001
Drinking, *n* (%)	356 (16.6)	169 (16.2)	69 (15.4)	118 (18.1)	0.438	194 (54.5)	162 (45.5)	0.002
Vegetable consumption, *n* (%)	1,912 (89.2)	919 (88.0)	408 (91.1)	585 (89.7)	0.192	1,218 (63.7)	694 (36.3)	< 0.001
Fruit consumption, *n* (%)	1,167 (54.4)	525 (50.3)	251 (56.0)	391 (60.0)	0.001	778 (66.7)	389 (33.3)	< 0.001
Physical activity, *n* (%)	1,599 (74.6)	750 (71.8)	343 (76.6)	506 (77.6)	0.016	1,108 (69.3)	491 (30.7)	< 0.001
Community activity, *n* (%)	1,703 (79.4)	813 (77.9)	356 (79.5)	534 (81.9)	0.136	1,130 (66.4)	573 (33.6)	< 0.001

TFI, Tilburg Frailty Indicator; SD, standard deviation; RMB, renminbi; ADL, activity of daily living. *P*-values are calculated with chi-square test (*n*, %) or students' t-test or ANOVA.

### Association between tea consumption and frailty

Table 2 shows the logistic regression results for the association between tea consumption and frailty. The results of model 1 indicated that occasional consumption of tea (OR = 0.74, 95%CI = 0.59–0.93, *P* < 0.01) and daily consumption of tea (OR = 0.60, 95%CI = 0.49–0.73, *P* < 0.001) were both associated with a lower prevalence of frailty. After controlling for sociodemographic characteristics and health status, the association was weakened, and only daily tea consumption (OR = 0.71, 95%CI = 0.56–0.90, *P* < 0.01) was associated with a lower prevalence of frailty. After further controlling for lifestyle factors, the association of tea consumption with frailty weakened, but the daily consumption of tea (OR = 0.73, 95%CI = 0.57–0.94, *P* < 0.05) was still associated with a lower prevalence of frailty. Furthermore, the study also found a linear relationship between tea consumption and frailty, indicating that higher tea consumption was associated with a lower prevalence of frailty (*P* for trend = 0.017).

**Table 2:** Associations between tea consumption and frailty among whole sample.

**Variables**	**Model 1**		**Model 2**		**Model 3**	
	**OR (95%CI)**	***P***	**OR (95%CI)**	***P***	**OR (95%CI)**	***P***
Daily consumption of tea	0.60 (0.49–0.73)	< 0.001	0.71 (0.56–0.90)	0.005	0.73 (0.57–0.94)	0.012
Occasionally consumption of tea	0.74 (0.59–0.93)	0.009	1.00 (0.77–1.30)	1.000	1.03 (0.79–1.35)	0.838
Age			1.03 (1.01–1.04)	< 0.001	1.03 (1.01–1.04)	0.001
Male			0.88 (0.71–1.09)	0.245	0.85 (0.68–1.06)	0.145
Rural residents			1.23 (0.97–1.55)	0.089	1.24 (0.97–1.58)	0.081
Married			0.51 (0.40–0.65)	< 0.001	0.52 (0.40–0.66)	< 0.001
Junior high school			0.74 (0.52–1.04)	0.086	0.83 (0.58–1.19)	0.318
High school and above			1.03 (0.79–1.33)	0.843	1.04 (0.80–1.35)	0.786
Income >3,000			0.64 (0.44–0.91)	0.014	0.67 (0.46–0.97)	0.032
Income 2,000–3,000			0.62 (0.49–0.78)	< 0.001	0.65 (0.51–0.83)	< 0.001
Chronic disease			2.38 (1.88–3.01)	< 0.001	2.10 (1.64–2.69)	< 0.001
ADL disability			3.73 (3.03–4.58)	< 0.001	3.15 (2.55–3.90)	< 0.001
Body mass index			1.00 (0.97–1.03)	0.917	1.00 (0.97–1.03)	0.935
Smoking					1.56 (1.24–1.95)	< 0.001
Drinking					1.33 (1.02–1.73)	0.037
Vegetable consumption					0.70 (0.50–0.97)	0.030
Fruit consumption					0.77 (0.62–0.95)	0.013
Physical activity					0.56 (0.44–0.71)	< 0.001
Community activity					0.60 (0.47–0.77)	< 0.001
*R* ^2^	0.009		0.181		0.214	

OR, odds ration; CI, confidence interval.

### Stratified analysis

Table 3 shows the results of a subgroup analysis of the association between tea consumption and frailty according to sex, age group, residence, and participation in community activities. In particular, daily consumption of tea was associated with a lower prevalence of frailty in men (OR = 0.69, 95%CI = 0.48–0.98, *P* < 0.05) but not in women (OR = 0.76, 95%CI = 0.54–1.08, *P* > 0.05). Meanwhile, the association between daily tea drinking and frailty was only statistically significant in older adults aged 60–79 years (OR = 0.70, 95%CI = 0.53–0.91, *P* < 0.01), but not in older adults aged ≥80 years. In addition, daily consumption of tea was significantly associated with a lower prevalence of frailty in rural residents (OR = 0.67, 95%CI = 0.50–0.91, *P* < 0.05), but not in urban residents (OR = 0.90, 95%CI = 0.59–1.37, *P* > 0.05). Moreover, this association was applicable only to older adults who regularly participated in community activities (OR = 0.76, 95%CI = 0.58–1.00, *P* = 0.05), and not to those who participated occasionally or rarely (OR = 0.73, 95%CI = 0.41–1.29, *P* > 0.05).

**Table 3:** Subgroup analyses of associations between tea consumption and frailty.

**Subpopulation**	**Occasionally consumption of tea**		**Daily consumption of tea**	
	**OR (95%CI)**	***P***	**OR (95%CI)**	***P***
By sex				
Male	1.07 (0.72–1.57)	0.750	0.69 (0.48–0.98)	0.037
Female	0.97 (0.65–1.43)	0.871	0.76 (0.54–1.08)	0.129
By age group				
60–79 years	0.96 (0.71–1.28)	0.769	0.70 (0.53–0.91)	0.008
≥80 years	1.58 (0.74–3.40)	0.240	0.96 (0.49–1.88)	0.909
By residence				
Urban residents	1.03 (0.65–1.63)	0.901	0.90 (0.59–1.37)	0.624
Rural residents	1.01 (0.72–1.41)	0.968	0.67 (0.50–0.91)	0.010
By community activity				
Regular participate	1.12 (0.83–1.50)	0.450	0.76 (0.58–1.00)	0.050
Occasional or seldom participate	0.74 (0.40–1.36)	0.328	0.73 (0.41–1.29)	0.278

## Discussion

We found that daily tea consumption was associated with a reduced prevalence of frailty in older adults. The results of this study confirm the findings of previous observational studies on the association between tea consumption and frailty in older adults (25–27). However, it should be noted that this association was heterogeneous regarding age, sex, residence, and participation in community activities. In particular, the association between tea consumption and frailty was significant among men, older adults aged 60–79, rural residents, and individuals who regularly participated in community activities.

We divided tea consumption into three categories to examine its association with frailty in this study. Only daily tea consumption was associated with a reduced prevalence of frailty, while occasional tea drinking was not. According to a study conducted in Japan, only consumption of high green tea was associated with a reduced risk of frailty (25). Two possible explanations can be offered for this association: physiological and psychosocial mechanisms. It is essential to first understand the pathophysiological pathways of frailty and its biomarkers before explaining the possible physiological mechanisms. Inflammation has previously been considered as one of the biological determinants of frailty, and biomarkers of frailty have been divided into four categories: inflammatory markers, oxidative stress, muscle protein turnover, and physical inactivity (35). It has been demonstrated that catechins, especially epigallocatechin gallate (EGCG), possess antioxidant, anti-inflammatory, and neuroprotective properties that can improve redox status at the tissue level, possibly preventing systemic structural damage (36, 37). In animal experiments, EGCG stimulates myogenic differentiation (38), inhibits aging-induced cardiac hypertrophy, fibrosis, and apoptosis (39), and reduces osteoclastogenesis (40). In addition, previous human intervention studies have also found that tea consumption protects against increased oxidative stress in older adults (41). These results support the beneficial biological effects of tea consumption on frailty. Moreover, in addition to its anti-inflammatory aspect, tea has long been associated with mood and performance enhancements. Previous study suggested that tea enhanced cognitive performance and psychological well-being (42). Tea consumption has increased during the COVID-19 pandemic, confirming the psychological effects of tea consumption as it may help relieve the higher levels of stress and disorder caused by the epidemic (43). In China and other Asian countries, consuming tea is an important way to reduce psychological stress, interact with others and participate in social activities (44, 45). A study of Chinese older adults indicated that tea drinking was associated with a lower risk of depression (46). Therefore, older adults who drink tea daily may be more likely to relieve stress and have a reduced risk of developing negative emotions. This may help reduce the risk of psychological frailty. In addition, people who drink tea regularly engage in more social activities and have wider social networks, reducing the risk of social frailty.

In this study, we further explored the heterogeneity in age, gender, residence, and participation in community activities on the association between tea consumption and frailty. The results showed that the negative relationship between daily consumption of tea and frailty was only observed in men, younger older adults, rural residents, and participants who regularly participated in community activities. These results are similar to those of previous studies that found a negative association between tea consumption and depressive symptoms, health status, and mortality in Chinese older adults (45, 47). Among those aged < 80 years, daily tea consumption was associated with a lower prevalence of frailty compared to those aged >80 years. A possible explanation is that frailty is an age-related disease; the oldest-old are more prone to frailty; therefore, the benefits of drinking tea may be difficult to highlight in the oldest-old. Previous research has also suggested that the health benefits of tea drinking may play a role in the early stages (under the age of 80) of health deterioration (45). In addition, we also found that the benefits of tea drinking on frailty were more pronounced in men and rural residents. This may be related to the high frequency of tea consumption among participants of these categories. Using univariate analysis, we found that men and rural residents consume more tea on a daily basis than women and urban residents; therefore, those who consume tea more frequently may experience greater health benefits. Finally, we found that tea consumption was negatively associated with frailty in older adults who regularly participated in community activities, but not in individuals who participated occasionally or rarely. As mentioned above, tea drinking was seen as one of the indicators of the social participation of older adults in China (48). Therefore, older adults who regularly participate in community activities are more likely to drink tea, which promotes social interactions and mental health; therefore, the association between tea drinking and frailty is more apparent. Since we did not find significant interactions between tea consumption and four of the factors on frailty, further research is warranted to explore the heterogeneities of these associations and their biological, psychological, and social mechanisms.

This study had several advantages. First, to our knowledge, this study is the first to explore the association between tea consumption and frailty among older adults in mainland China. In particular, we comprehensively evaluated the frailty of older adults from three levels: physical, psychological, and social, rather than focusing solely on physical frailty. Second, we controlled for a set of covariates in the regression to make the association between tea consumption and frailty more robust. In short, this study provides research evidence from China to explore the association between tea drinking and frailty, thus further enriching the literature on the health benefits of drinking tea. Considering the availability of tea, this study suggests that promoting tea consumption in older adults may be an effective measure to help reduce the prevalence of frailty. However, it must be noted that the results of this study should be interpreted with caution, as the underlying mechanisms of action of tea on health, remain unclear. The statistical associations in this study did not demonstrate a clinical effect of tea drinking on decrease of frailty in older adults.

This study also had several limitations. First, the cross-sectional study design made it impossible to infer a causal relationship between tea consumption and frailty. In the future, it will be necessary to use longitudinal studies to examine the association between the two and to use interventional studies to establish a causal relationship. Second, tea consumption in this study was self-reported, and the exact amount consumed could not be determined due to a lack of data, which may introduce both recall and measurement bias. In addition, we did not analyze the association between different types of tea and frailty. Further research should be conducted on the association of different tea types with frailty. Green tea and fermented tea contain different levels of tea polyphenols; thus, their associations with frailty may also differ. Third, when explaining the association between tea consumption and frailty, we mainly demonstrated the aspect of inflammation. However, we did not collect inflammatory biomarkers in the survey. Future studies should further focus on the levels of inflammation in the investigated individuals and a rather healthy and active lifestyle that may be associated with tea drinking. This may help further clarify the mechanism of the association between healthy lifestyles, including tea consumption and frailty, to provide a higher level of scientific evidence for the prevention of frailty.

## Conclusions

Tea consumption is associated with a lower prevalence of frailty among older adults, especially those men, older adults aged 60–79, rural residents, and individuals who regularly participated in community activities. Further longitudinal and experimental studies are needed to determine the causal relationship between tea consumption and frailty.

## Data availability statement

The raw data supporting the conclusions of this article will be made available by the authors, without undue reservation.

## Ethics statement

The studies involving human participants were reviewed and approved by Medical Ethics Committee of Xiangya School of Public Health, Central South University (identification code: XYGW-2020-101). The patients/participants provided their written informed consent to participate in this study.

## Author contributions

SL, FL, and YYa contributed to the study design. SL analyzed the data and drafted the manuscript. GC and YYi conducted data collection and gave comments on the draft. FL and YYa revised the draft. All authors read and approved the final manuscript.

## Conflict of interest

The authors declare that the research was conducted in the absence of any commercial or financial relationships that could be construed as a potential conflict of interest.

## Publisher's note

All claims expressed in this article are solely those of the authors and do not necessarily represent those of their affiliated organizations, or those of the publisher, the editors and the reviewers. Any product that may be evaluated in this article, or claim that may be made by its manufacturer, is not guaranteed or endorsed by the publisher.
